# Candidate autoantigens identified by mass spectrometry in early rheumatoid arthritis are chaperones and citrullinated glycolytic enzymes

**DOI:** 10.1186/ar2644

**Published:** 2009-03-10

**Authors:** Vincent Goëb, Marlène Thomas-L'Otellier, Romain Daveau, Roland Charlionet, Patrice Fardellone, Xavier Le Loët, François Tron, Danièle Gilbert, Olivier Vittecoq

**Affiliations:** 1 Department of Rheumatology and Inserm Unit 905, IFRMP 23, Institute for Biomedical Research, University of Rouen, Rouen University Hospital, Rouen 76031 cedex, France; 2 Immunology Laboratory and Inserm Unit 905, IFRMP 23, Institute for Biomedical Research, University of Rouen, Rouen University Hospital, Rouen, 76031 cedex, France; 3 Rheumatology Department, Amiens University Hospital, Amiens 80054, France

## Abstract

**Introduction:**

The aim of our study was to identify new early rheumatoid arthritis (RA) autoantibodies.

**Methods:**

Sera obtained from 110 early untreated RA patients (<6 months) were analyzed by western blot using HL-60 cell extract, separated on one-dimensional and two-dimensional gel electrophoresis (1-DE, 2-DE). Sera from 50 healthy blood donors and 20 patients with non-RA rheumatisms were used as controls for 1-DE and 2-DE, respectively. The immunoreactive proteins were identified by MALDI-TOF mass spectrometric analysis and the presence of potential sites of citrullination in each of these proteins was evaluated. FT-ICR mass spectrometry was used to verify experimentally the effect of citrullination upon the mass profile observed by MALDI-TOF analysis.

**Results:**

The 110 1-DE patterns allowed detection of 10 recurrent immunoreactive bands of 33, 39, 43, 46, 51, 54, 58, 62, 67 and 70 kDa, which were further characterized by 2-DE and proteomic analysis. Six proteins were already described RA antigens: heterogeneous nuclear ribonucleoprotein A2/B1, aldolase, α-enolase, calreticulin, 60 kDa heat shock protein (HSP60) and BiP. Phosphoglycerate kinase 1 (PGK1), stress-induced phosphoprotein 1 and the far upstream element-binding proteins (FUSE-BP) 1 and 2 were identified as new antigens. Post-translational protein modifications were analyzed and potentially deiminated peptides were found on aldolase, α-enolase, PGK1, calreticulin, HSP60 and the FUSE-BPs. We compared the reactivity of RA sera with citrullinated and noncitrullinated α-enolase and FUSE-BP linear peptides, and showed that antigenicity of the FUSE-BP peptide was highly dependent on citrullination. Interestingly, the anti-cyclic citrullinated peptide antibody (anti-CCP2) status in RA serum at inclusion was not correlated to the reactivity directed against FUSE-BP citrullinated peptide.

**Conclusions:**

Two categories of antigens, enzymes of the glycolytic family and molecular chaperones are also targeted by the early untreated RA autoantibody response. For some of them, and notably the FUSE-BPs, citrullination is involved in the immunological tolerance breakdown observed earlier in RA patients. Autoantibodies recognizing a citrullinated peptide from FUSE-BP may enhance the sensibility for RA of the currently available anti-CCP2 test.

## Introduction

Rheumatoid arthritis (RA) is a disabling autoimmune and inflammatory disease affecting between 0.3% and 1% of the population in developed countries. The heterogeneity of disease manifestations and the clinical course constitutes a challenge for clinicians to predict the severity of the disease and to choose the appropriate therapy early. The autoimmune response appears early, often prior to the apparition of clinical symptoms, and leads to the production of various autoantibodies (autoAb) easily detectable in serum. These autoAb help to understand pathological mechanisms and constitute biological markers of the disease [1].

Furthermore, we recently assessed the contribution of several genetic markers (*HLA*-shared epitope, *TNFR2 *196R and *PTPN22 *1858T alleles) for RA diagnosis and found that the autoimmune markers (rheumatoid factors and anti-citrullinated protein antibodies (ACPA)) were the best parameters to predict RA diagnosis precociously [2]. ACPA have been originally described as anti-keratin autoAb [3], anti-perinuclear autoAb [4] and then as anti-filaggrin autoAb [5]. As a matter of fact, ACPA recognize the deiminated form of filaggrin [6] and can be detected using several peptide sequences in which arginine is substituted with citrulline flanked by neutral amino acids as antigens [7]. Whether filaggrin is the true autoantigen of ACPA is unlikely since it is exclusively expressed in epithelial cells, and other citrullinated proteins – such as fibrinogen [8], vimentin [9], enolase [10], collagen type I [11], fibronectin [12], a translational initiation factor [13] and even a viral protein, EBNA-1 [14] – have been shown to be the target of the autoimmune response. The deimination of proteins is mediated by peptidylarginine deiminase (PADI) and occurs notably during cell death and oxidative stress [15,16], both events observed in RA synovium.

Proteomic technologies rely on the ability to separate a complex mixture of proteins and to identify them by different methods, in particular mass spectrometry (MS) using matrix-assisted laser desorption/ionization–time of flight (MALDI-TOF) analysis. Separated proteins are digested with enzymes such as trypsin, then the peptide mass fingerprinting is used to search sequence databases and to identify proteins that match the observed fragment pattern. The identification of protein biomarkers specific for inflammatory diseases, and particularly for RA [17], may therefore provide highly sensitive diagnosis tools and a better understanding of the mechanisms underlying these disorders.

The present study was performed in order to identify new proteins targeted by the early untreated RA autoimmune response and their potential post-translational modifications (PTMs) that could lead to the production of autoAb. These proteins were identified after separating HL-60 extracts by two-dimensional gel electrophoresis (2-DE) and localizing the antigens by immunoblotting with patient sera. Protein spots were analyzed by MALDI-TOF mass spectrometric analysis. In each of the different proteins highlighted, the presence of potential sites of citrullination was investigated. Finally, the reactivity of RA sera's autoAb against some citrullinated peptides corresponding to the citrullinated antigens was assessed by Luminex assay.

## Materials and methods

### Patients

Serum samples were collected from 110 RA patients among the 314 very early arthritis patients recruited in the Very Early Arthritis (VErA) cohort [18], including RA, non-RA well-defined rheumatic diseases and undifferentiated polyarthritis. Briefly, patients of the VErA cohort were required to have swelling of at least two joints that had persisted for longer than 4 weeks but had been evolving for less than 6 months, and who had not received disease-modifying anti-rheumatic drugs and/or steroid therapy before inclusion. All participants were European Caucasians.

The Committee for Protection of Persons Participating in Biomedical Research of Rouen, France, approved the protocol. All of the patients gave their informed consent for the study (French law 88-1138; 20 December 1988). RA patients were evaluated and classified using the American College of Rheumatology 1987 criteria for RA [19] at 2 years of follow-up. Only sera collected at the time of inclusion (median duration of the symptoms, 4 months) were analyzed in the present study. Serum samples collected from 50 healthy blood donors and 20 patients with non-RA rheumatic diseases from the VErA cohort were used as controls for one-dimensional gel electrophoresis (1-DE) and 2-DE, respectively.

### Preparation of cell lysates

Since most RA autoantigens are ubiquitously expressed and myeloid cells are the dominant cell type present in the rheumatoid joint, we selected HL-60, a human promyelocytic leukemia cell line (American Collection of Cell Culture, Rockville, MD, USA), for the present study. The HL-60 cell line was frozen in FCS supplemented with 10% dimethyl sulfoxide, and was kept in liquid nitrogen. In order to obtain cell lysates, HL-60 cells were thawed and grown in a large volume of complete medium, RPMI 1640, sodium pyruvate 10%, FCS 10%, penicillin–streptomycin 1% at 37°C in a humidified atmosphere (5% CO_2_), then centrifuged, washed twice with sucrose, and the pellet was frozen at -80°C until use. Proteins were extracted according to Görg and colleagues [20], by precipitation in organic solvent before being lysed in 9 M urea containing 2% 3-[(3-Cholamidopropyl)dimethylammonio]-1-propanesulfonate (CHAPS), 20 mM dithiothreitol (DTT) and protease inhibitor cocktail (Sigma-Aldrich, St Louis, MO, USA). The lysate was sonicated (Vibra Cell; Bioblock Scientific, Illkirch, France), centrifuged at 15,000 rpm for 30 min at 4°C, and frozen at -80°C.

### One-dimensional gel electrophoresis and western blotting

HL-60 cells proteins were separated by 1-DE on 4% to 12% precast Bis–Tris NuPAGE gels, using MOPS running buffer (Invitrogen, Carlsbad, CA, USA). After separation, proteins were transferred onto nitrocellulose membranes (Hybond™-c extra; GE Healthcare Life Sciences, Piscataway, NY, USA) and stained with Ponceau red (Sigma-Aldrich). Membranes were cut and the strips were saturated with PBS–5% dry milk, were incubated with patient sera (1:100 dilution), were incubated with biotinylated conjugated mouse monoclonal anti-human IgG (Fc) (Southern Biotechnology Associates Inc., Birmingham, AL, USA), were incubated with alkaline phosphatase-conjugated streptavidin (CALTAG; Invitrogen), and were revealed with NBT/BCIP (Roche Applied Science, Indianapolis, IN, USA). Each step was followed by three washes with PBS/Tween 0.05% buffer.

### Data-processing analysis

One-dimensional immunoblotting patterns, given by sera from 110 RA patients and 50 healthy blood donors, were analyzed with the Image Master TotalLab software (GE Healthcare Life Sciences), in order to identify the various protein patterns after background removal, and to measure the migration distance and expression intensity of each band. Perl and R scripts were developed for standardization of the molecular weight (MW) and the expression level. Selected serum protein patterns were then studied in further detail by 2-DE.

### Two-dimensional gel electrophoresis

RA and non-RA control sera were analyzed by western blot using 2-DE membranes. Proteins were focused at 20°C, with 11 cm immobilized pH 3 to 10 gradient IPG ReadyStrips (BIO-RAD Laboratories, Hercules, CA, USA) that were incubated for 16 hours in 200 μl protein extract mixed with rehydration buffer (8 M urea, 2% CHAPS, 1% DTT, trace of bromophenol blue, 0.2% Biolyte carrier ampholytes 3 to 10; BIO-RAD Laboratories). The Protean IEF cell (BIO-RAD Laboratories) was used with fast-voltage ramping at a maximum voltage of 6,000 V for 20 hours. After the first dimension run, the strips were equilibrated by incubation in 6 M urea, 0.375 M Tris–HCl, pH 8.8, 2% SDS, 20% glycerol, 2.5% (w/v) DTT 10 ml per strip for 20 minutes at room temperature, followed by an incubation for 30 minutes in the same buffer but in which DTT was replaced by 2.5% (w/v) iodoacetamide. Strips were then placed on the top of 4% to 12% Criterion™ XT precast gels (11 cm × 8 cm × 1 mm) (BIO-RAD Laboratories) and migrated constantly at 200 V until the bromophenol blue dye front had reached the bottom of the gel. The BenchMark™ prestained protein ladder (Invitrogen) was used as the MW standard in the second dimension step. In some experiments, this ladder was replaced by the protein extract in order to visualize both 1-DE and 2-DE protein patterns on the same membrane. Finally, gels were either stained with Coomassie brilliant blue G250 (Sigma-Aldrich) or were electroblotted for 1 hour onto nitrocellulose membranes, and western blotting analyses were performed as previously described. G250-stained 2-DE gels were scanned using a densitometer, and images were obtained with digitalization software (2-D Phoretix, Alphelys Plaisir, France). Immunoreactive spots were selected by comparing the immunoblotted replica with G250-stained gels.

### Protein identification

The immunoreactive spots were excised from polyacrylamide gels with Ettan Spot Picker (GE Healthcare Life Sciences) and were digested by proteomics-grade trypsin (Sigma-Aldrich) with Ettan Digester (GE Healthcare Life Sciences). After digestion, peptides were extracted with 50% acetonitrile, 0.1% trifluoroacetic acid and mixed on the MALDI-TOF target (Applied Biosystems, Foster City, CA, USA) with an equal matrix volume of 7.5 mg/ml α-cyano-4-hydroxy cinnamic acid (LaserBio Labs, Sophia Antipolis, France) saturated with 50% acetonitrile, 0.1% trifluoroacetic acid.

Samples were analyzed by mass spectrometry with a MALDI-TOF Voyager-DE™ PRO (Applied Biosystems) using a delayed ion extraction and ion mirror reflector mass spectrometer. The instrument settings were: reflector mode with positive polarity, 100 nanosecond delay extraction time, 70% to 80% grid voltage and 20,000 V accelerating voltage. Laser shots at 500 per spectrum were used to acquire one spectrum with a mass range from 700 to 4,000 Da. External calibration was carried out using the Proteomix–Peptide calibration Mix4 (LaserBio Labs). Spectra were accumulated manually from different acquisitions to improve resolution and the signal-to-noise ratio.

The tools used to identify proteins from peptide mass fingerprinting data were Aldente and FindMod [21,22], which can be found on the Expasy server [23]. By looking over differences between experimentally determined and theoretical peptide masses from a specified protein, FindMod permits one to discover PTMs and to make predictions as to what amino acid in the peptide is likely to carry the modification. Several possibilities were often suggested that stand within the selected mass tolerance, but most of them could be eliminated using a manual spectrum recalibration. The peptides were generated by trypsin that cleaves proteins at the C-terminal side of K or R. The number of missed cleavages allowed was set to 1 for Aldente and was set up to 3 for FindMod analysis. Several chemical modifications occurring during the separation process were taken into account in Aldente and FindMod analysis: carboxyamidomethyl cysteine due to the action of iodoacetamide on cysteine residues, propionamide cysteine that is an acrylamide adduct to cysteine, and methionine sulfoxide linked to the presence of ammonium persulfate in the gel.

### Characterization of citrullination by mass fingerprinting

After the identification of immunoreactive proteins with the Aldente program, the corresponding spectra were further examined in order to detect the presence of several types of PTM of discrete mass. The FindMod and FindPept programs (Expasy server [23]) were used for looking at mass differences between experimentally determined peptide masses and theoretical peptide masses. When a mass difference corresponding to a known PTM was observed, rules were applied that examine the sequence of the peptide of interest and make predictions as to which amino acid in the peptide was likely to carry the modification. These rules are included either in the FindMod and FindPept programs or in the various tools and software for PTMs found on the Expasy server [23] (for instance, NetPhos or NetAcet).

In our study, a particular attention was paid to citrullination, a PTM occurring on arginine residues. Several rules were applied: for one citrullinated arginine, the peptide theoretical mass increase is 0.98 Da and the modified peptide, losing one amino group, becomes more acidic [24]; citrullinated arginine residues are not likely to be cleaved by trypsin, so that a minimum number of one missed cleavage must be specified and a peptide that includes a C-terminal citrullinated arginine must be rejected; and in a biological sample, only a fraction of a given protein may be citrullinated at a specific site. Because of the several PTMs occurring on a given protein, this protein was generally found on a two-dimensional map as a train of spots. A spot separated by two-dimensional gel may thus contain the same protein with several PTMs. Consequently, a citrullinated peptide proposed by FindMod should incite one to search for the modified and unmodified peptides in the spectra of this protein, both peptides differing only by 0.98 Da generating an unusual isotopic mass cluster.

Otherwise, to verify these specifications for the characterization of citrullination by mass fingerprinting, we deiminated *in vitro *aldolase purified from rabbit muscle (Sigma-Aldrich) with PADI from rabbit skeletal muscle (Sigma-Aldrich). Then 25 μg purified aldolase were incubated with 0.2 units PADI in buffer containing 0.1 M Tris–HCl, pH 7.4, 10 mM CaCl_2_, 5 mM DTT, at 37°C for 90 minutes. The citrullination processes were followed by 2-DE analysis, enzymatic digestion of the various citrullinated aldolase obtained and analysis of the peptides by MALDI-TOF MS and by Fourier transform ion cyclotron resonance (FT-ICR) mass spectrometer.

### Fourier transform ion cyclotron resonance mass spectrometer

The peptide sequence spectra were obtained using nanochromatography (Ultimate LC system, Dionex; LC-Packings, Amsterdam, the Netherlands) online with an Apex Qe 9.4 T FT-ICR mass spectrometer (Bruker Daltonics, Bremen, Germany). Starting from a volume of 1 μl peptide solution, peptides were desalted and concentrated on a C18 preconcentration column (5 cm × 300 μm) and separated on a Pepmap C18 column (15 cm × 75 μm) at 200 nl/min solvent flow. The elution was performed using gradients of solvent A (95% H_2_O, 5% acetonitrile, 0.1% HCOOH) and solvent B (20% H_2_O, 80% acetonitrile, 0.1% HCOOH): 15 minutes in 100% solvent A, then solvent B was increased to 100% in 130 minutes, then kept at 100% for 15 minutes, and then finally solvent B decreased to 0% in 5 minutes. The column was allowed to equilibrate for 15 minutes before another run.

The FT-ICR mass spectrometer is equipped with a nano-electrospray source. Detection was carried out in the positive mode. A potential of 1.7 kV was applied on the needle. The time cycle of an experiment for each spectrum, including accumulation, transfer, excitation, detection and quench, ran for approximately 3 seconds. In detail, ions were accumulated for 1 second in the hexapole, and 2 seconds in the quadrupole collision cell; 0.0016 seconds was set for optics transfer and 0.01 seconds for the electronic dwell time. The detection parameters were broadband detection, 512 K acquisition size, and start mass at *m/z *200 leading to 0.5243 seconds transient duration allowing theoretical resolution of 190,000 at *m/z *400. For the liquid chromatography–MS run, the quadrupole was not resolving and set at *m/z *350 and the collision energy set at 1.5 eV. For liquid chromatography–MS/MS runs, the quadrupole was resolving and set at the required mass *m/z *824.2 and the collision energy set at 28.5 eV. The mass window of the selecting quadrupole was 2 mass units. Spectra were annotated using the fragment algorithm in the Distiller software from Matrixscience (Matrix Science Ltd., London, UK), which allows introducing the required modifications (deamidation, citrullination) on specific amino acids.

### Detection of citrullinated proteins and deimination *in vitro*

After transfer, the membranes were saturated with blocking buffer and were incubated with rabbit immunoaffinity purified IgG anti-citrulline (Upstate Biotechnology, Lake Placid, NY, USA). Biotinylated-goat anti-rabbit and IRDye 800-conjugated streptavidin were used as secondary antibodies and were visualized using the Odyssey™ Infrared Imaging system (LI-COR Biosciences, Lincoln, NE, USA) according to the manufacturer's protocol with minor modifications. In some experiments, membranes were incubated with 2 units PADI from rabbit skeletal muscle (Sigma-Aldrich) in buffer containing 0.1 M Tris–HCl, pH 7.4, 10 mM CaCl_2_, 5 mM DTT, overnight at 37°C.

### Anti-citrullinated protein antibody detection

The presence of ACPA was detected using anti-cyclic citrullinated peptide antibody (anti-CCP2) commercially available kits (EuroImmun, GMBH, GroB Grönau, Germany). In the present study, we have considered both ACPA positivity (threshold, 10 arbitrary units) and the level measured during the inclusion.

### Anti-peptide antibody detection

We designed six deiminated peptides using both linear citrullinated peptides and CCPs. Their sequences were determined from those identified by MALDI-TOF MS analysis. In addition, we introduced six histidines for coupling to LiquiChip Ni-NTA beads (LiquiChip NiNTA; Qiagen, SA, Courtaboeuf, France). For cyclic peptides, cysteine residues were added at each extremity to create a disulfide bridge. All of the peptides were purchased from Millegen (Labege, France). Ni-NTA beads were incubated with peptides overnight. For antibody detection, beads mixed together were added to patient sera diluted 1:100 and were incubated at room temperature for 30 minutes. After a wash cycle, biotin-conjugated anti-human IgG (Southern Biotechnological) was added for 30 minutes followed by streptavidin-PE (Qiagen SA) for 15 minutes. The bead mixture was analyzed by passing through the detector of a Bio-Plex system (BIO-RAD, Marnes-la-Coquette, France) that identifies the beads based on the fluorescence of the dyes. The amount of antibody bound to the bead was determined by the fluorescence of PE. The fluorescence intensity values obtained with noncitrullinated peptides were subtracted from those observed with the corresponding citrullinated peptides; a difference above 100 units of fluorescence intensity was considered positive.

### Statistical analysis

Wilcoxon nonparametric and Student parametric tests were used to determine whether the presence and titer of ACPA were associated with the presence of antibodies directed against the 1-DE-separated polypeptide bands, and whether the presence of antibodies directed against the highlighted antigens was associated with that of antibodies directed against corresponding synthetic citrullinated peptides. We also assessed whether the presence of antibodies directed against synthetic citrullinated peptides was correlated with the presence of ACPA (anti-CCP2 test) at inclusion. For all tests, *P *< 0.05 was considered statistically significant.

## Results

### Detection of autoantibodies in RA patient sera by western blot analysis

**Figure 1:**
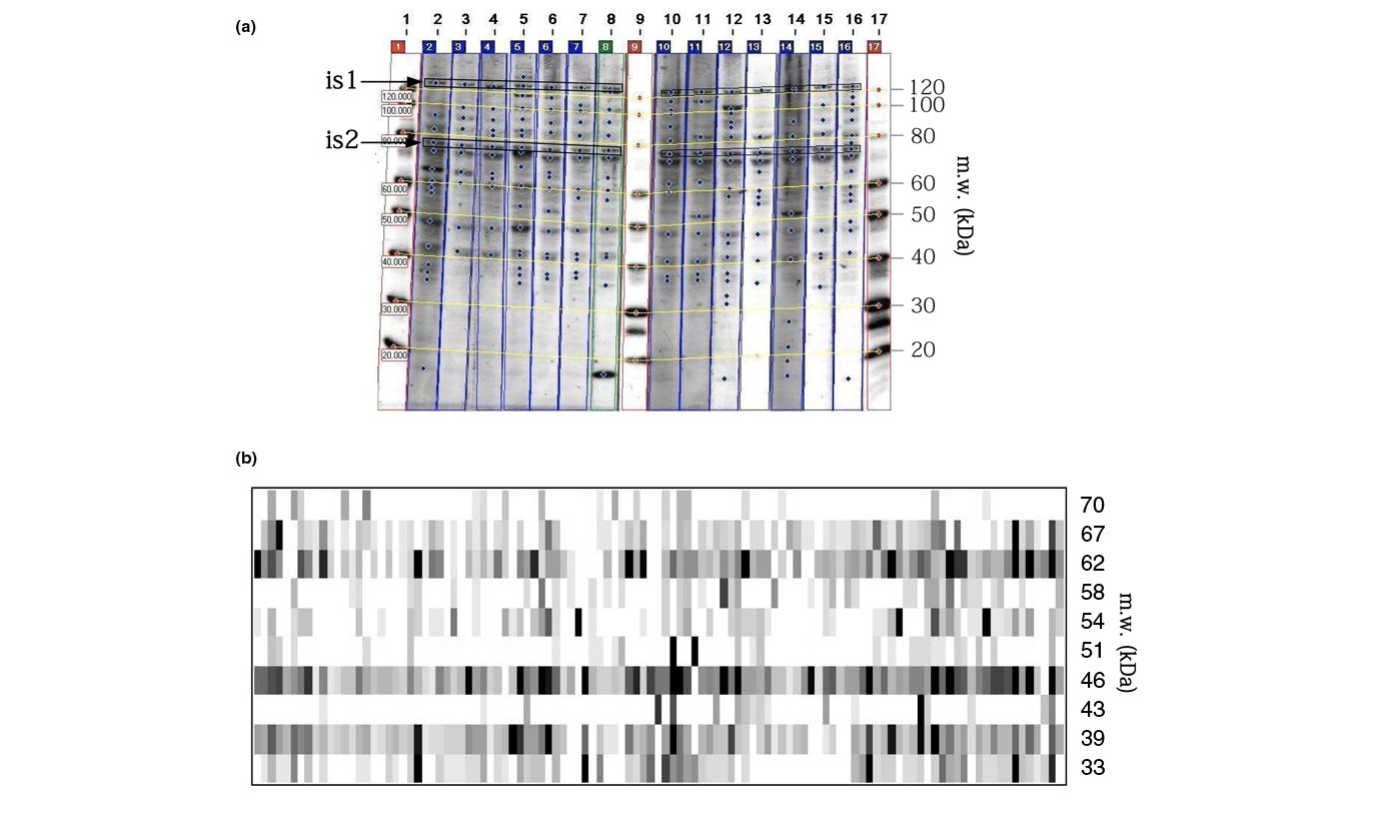
Detection of autoantibodies in rheumatoid arthritis patient sera. Autoantibodies in rheumatoid arthritis (RA) patient sera were detected by western blot analysis using HL-60 cell extract as the substrate. **(a) **Example of one-dimensional gel electrophoresis western blot analysis with Imagemaster totalLab software to determine the molecular weights (m.w.) of different bands using an internal standard (is1 and is2) that correspond to 120-kDa and 80-kDa proteins revealed by alkaline phosphatase-conjugated streptavidin. These bands were used for standardization between the different membranes. **(b) **Virtual blot of the 110 RA patient sera. The m.w. of the bands are indicated on the right-hand side of the figure. Each vertical lane corresponds to different RA patient sera.

As the first step of new disease-specific autoantibody detection, each of the 110 sera obtained at inclusion from RA patients recruited into the VErA cohort was studied by western blot analysis on HL-60 cell extract separated on 1-DE. All of the membranes were analyzed by scanning densitometry and the quantification of bands was normalized using internal standards for each band (Figure 1a). Among the 110 patterns, compared within the interval of 33 to 70 kDa, 10 bands of 33, 39, 43, 46, 51, 54, 58, 62, 67 and 70 kDa were recognized by 31, 37, 4, 53, 9, 25, 11, 40, 14 and 9 RA sera, respectively.

Table 1 presents the reactivity of the 110 RA sera that was compared with that of 50 control sera obtained from healthy blood donors. Nine of the latter (9/50) bound to the p46 polypeptide, which corresponds to α-enolase (see below). Forty-one healthy sera (82%) were therefore clearly negative with respect to α-enolase recognition. A virtual representation of the RA patterns is shown in Figure 1b.

**Table 1:** Reactivity of rheumatoid arthritis and healthy control sera with HL-60-derived proteins

	HL-60-derived polypeptides									
	
	p33	p39	p43	p46	p51	p54	p58	p62	p67	p70
Rheumatoid arthritis sera (n = 110)	31	37	4	53	9	25	11	40	14	9
Control sera (n = 50)	0**	0**	0	9**	0	0**	3	15*	0**	6

Data expressed as the number of sera that bind the different polypeptides by western blot analysis. *0.002 <*P *< 0.005, **P < 0.0004.

### Identification of immunoreactive spots

**Figure 2:**
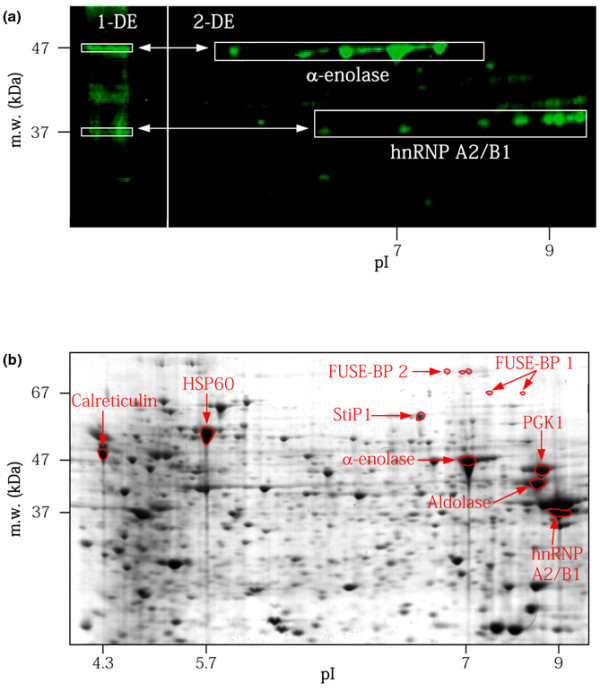
Identification of proteins contained in the HL-60 cell map and bound by rheumatoid arthritis sera. **(a) **Western blot analysis of a rheumatoid arthritis (RA) serum recognizing both 50-kDa and 33-kDa proteins, using the Odyssey™ Infrared Imaging system. HL-60 cell lysates were separated by two-dimensional gel electrophoresis (2-DE) using 11 cm readyStrip™ IPG strips (pH 3 to 10, nonlinear) in the first dimension and precast Criterion XT Bis-Tris gels (4% to 12% resolving gels, IPG+1 well) in the second dimension. The protein extract was put in the one-dimensional well instead of the molecular weight (m.w.) to visualize both the one-dimensional and two-dimensional patterns. The proteins were electroblotted onto nitrocellulose membranes, then incubated with RA sera. **(b) **Immunoreactive spots were identified by mass spectrometry with a matrix-assisted laser desorption/ionization–time of flight Voyager-DE™ using 2-DE-separated HL-60 protein maps, stained by Coomassie brilliant blue G250. 1-DE, one-dimensional gel electrophoresis; FUSE-BP, far-upstream element-binding protein; hnRNP A2/B1, heterogeneous nuclear ribonucleoprotein A2/B1; HSP60, 60 kDa heat shock protein; PGK1, phosphoglycerate kinase 1; StiP1, stress-induced phosphoprotein 1.

To elucidate the nature of proteins contained in these bands, we performed target-oriented proteomics using the 2-DE-separated-HL60 protein map followed by western blot analysis with RA sera selected on the basis of their 1-DE pattern. Fifty RA sera were analyzed by two-dimensional PAGE to simultaneously visualize 1-DE bands and 2-DE immunoreactive spots on the same membrane. An example of a RA serum recognizing both α-enolase and heterogeneous nuclear ribonucleoprotein A2/B1 is shown in Figure 2a. All of the immunoreactive spots were excised from polyacrylamide gel and digested by trypsin. The peptides were analyzed by MS and were analyzed using the Aldente and FindMod tools. The comparison of the mass spectra obtained for each spot with those contained in the Swiss-Prot database allowed us to identify with high probability the immunoreactive proteins. All of the identifications of immunoreactive spots were obtained from three separate experiments.

Table 2 presents the identities of the 10 immunoreactive spots with their Aldente and *Z *scores, and summarizes all of the hits (that is, the peak matching a theoretical peptide) and the coverage found with both Aldente and FindMod software. We therefore identified heterogeneous nuclear ribonucleoprotein A2/B1 at 33 kDa, fructose-biphosphate aldolase A (aldolase) and phosphoglycerate kinase 1 (PGK1) at 39/43 kDa, α-enolase and calreticulin at 46/51 kDa, 60 kDa heat shock protein (HSP60) and stress-induced phosphoprotein 1 at 58/62 kDa, and far upstream element-binding proteins 1 and 2 (FUSE-BP1 and FUSE-BP2) and BiP, also named GRP78, at 67/70 kDa (Figure 2b). Since 2-DE separates proteins with identical MW but different isoelectric points, several antigens were identified for a given MW.

**Table 2:** Identities of immunoreactive spots from MALDI-TOF spectra using the Aldente and FindMod tools

Protein	Swiss-Prot number	Theoretical MW (Da)/pI	Hits^a^		Coverage (%)^b^		Aldente score^c^	Aldente *Z *score^d^
					
			Aldente	FindMod	Aldente	FindMod		
Heterogeneous nuclear ribonucleoprotein A2/B1	[Swiss-Prot:P22626]	37,430/9.0	17	21	51	51	49.54	655.1
Aldolase	[Swiss-Prot:P04075]	39,288/8.4	22	60	69	86	44.36	814.8
Top of form 1 phosphoglycerate kinase 1	[Swiss-Prot:P00558]	44,728/8.3	15	25	40	54	21.20	416.8
α-Enolase	[Swiss-Prot:P06733]	47,169/7.0	12	33	39	59	16.19	208.2
Calreticulin	[Swiss-Prot:P27797]	48,142/4.3	16	51	36	51	20.14	99.5
Heat shock protein 60	[Swiss-Prot:P10809]	61,055/5.7	26	41	52	64	107.28	1389
Stress-induced phosphoprotein 1	[Swiss-Prot:P31948]	62,638/6.4	19	31	38	49	36.04	898.2
FUSE-BP1	[Swiss-Prot:Q96AE4]	67,474/7.2	15	23	30	35	41.78	402.7
FUSE-BP2	[Swiss-Prot:Q92945]	72,708/8.2	12	23	22	25	13.64	171.4
BiP	[Swiss-Prot:P11021]	72,334/5.1	25	34	44	47	94.41	1616.3

FUSE-BP, far-upstream element-binding protein; MALDI-TOF, matrix-assisted laser desorption/ionization–time of flight; MW, molecular weight; pI, isoelectric point. ^a^A hit is an experimental peak matching a theoretical peptide. ^b^The coverage is the number of amino acids present in at least one peptide/the number of amino acids of the protein. ^c^The Aldente tool gives a score to each identified protein. The parameters selected in these scores are the number and the intensity of hits, the number of missed cleavages, the C-terminal amino acid, the chemical modifications and, at the protein level, the coverage of the identified peptides on the sequence. The score of the proteins identified in this study are largely greater than the score of the best random protein. ^d^The *Z *score is the number of standard deviations for a given score from the mean random score.

Among the 20 sera from non-RA rheumatic diseases of the VErA cohort, two sera weakly recognized α-enolase. These two sera were obtained from patients who had undifferentiated arthritis. Except for α-enolase, the other immunoreactive spots were never bound by any autoAb.

### Characterization of citrullination by mass fingerprinting

After the identification of immunoreactive proteins with the Aldente program, the corresponding spectra were further examined in order to detect the presence of several types of PTMs of discrete mass. Among the PTMs observed for most of proteins, we focused our attention on potentially deiminated peptides – we found that seven out of the 10 proteins (aldolase, α-enolase, PGK1, calreticulin, HSP60, FUSE-BP1 and FUSE-BP2) possessed such peptides (Table 3).

**Table 3:** Potentially deiminated peptides from MALDI-TOF spectra using the Aldente and FindMod tools

Protein	Sequence of peptides	Theoretical molecular weight (Da)	Position	Missing cleavage
Aldolase	KDGADFAKWR_citr_CVLK	1,856.921	139 to 152	3
Phosphoglycerate kinase 1	ALESPER_citr_PFLAILGGAK	1,769.979	199 to 215	1
α-Enolase	YNQLLR_citr_IEEELGSKAK	1,892.012	406 to 421	2
Calreticulin	DKQDEEQR_citr_LK	1,336.623	359 to 368	2
Heat shock protein 60	KDR_citr_VTDALNATR	1,360.718	418 to 429	2
	R_citr_GVMLAVDAVIAELKK	1,729.988	142 to 157	2
FUSE-BP1	VPDGMVGFIIGR_citr_GGEQISR	2,003.038	106 to 124	1
FUSE-BP2	TSMTEEYRVPDGMVGLIIGRGGEQINK (one of these R is citrullinated)	2,967.475	143 to 169	2

FUSE-BP, far-upstream element-binding protein; MALDI-TOF, matrix-assisted laser desorption/ionization–time of flight.

### *In vitro *citrullination of aldolase

**Figure 3:**
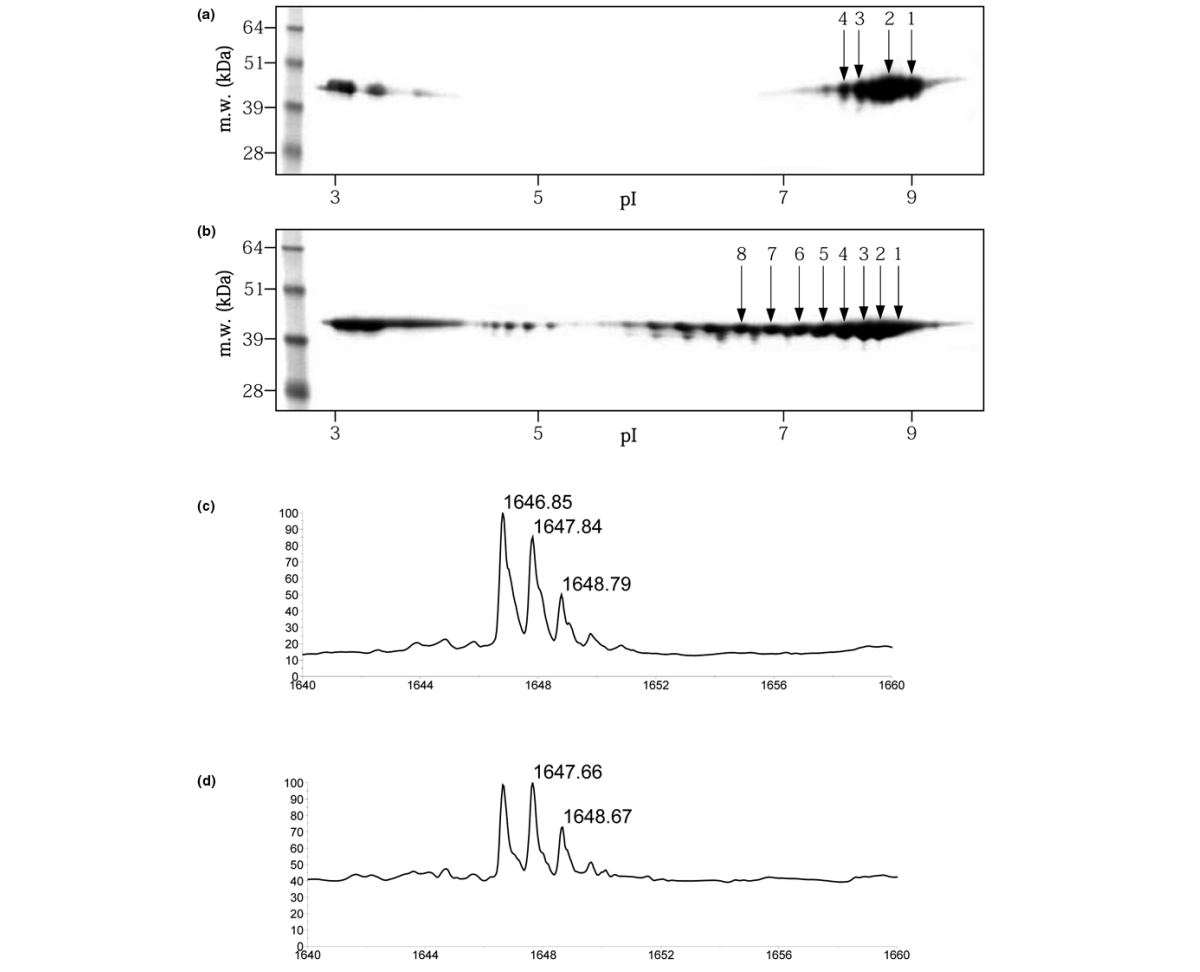
Two-dimensional gel electrophoresis maps of native and citrullinated rabbit aldolase. Two-dimensional gel electrophoresis maps of **(a) **native rabbit aldolase and **(b) **citrullinated rabbit aldolase. Mass spectra of **(c) **digested spot 1 and **(d) **digested spot 5. The isotope clusters correspond to the peptide RLQSIGTENTEENR with a mass of 1,646.809 Da for the peptide without post-translational modification and of 1,660.825 for the methylated peptide. The intensity increase observed for the second peak of the isotopic clusters in (d) is linked to the rate of acidification of the protein; this indicates the citrullination process. Beyond the fifth spot in (b), the rabbit aldolase is too citrullinated for the peptide RLQSIGTENTEENR to be seen after the trypsin digestion. m.w., molecular weight; pI, isoelectric point.

To verify experimentally the effect of citrullination upon the mass profile observed by MALDI-TOF analysis, we proceeded with the *in vitro *deimination of aldolase purified from rabbit muscle. Figure 3 shows the 2-DE maps of native and citrullinated aldolase, respectively. The observed acidification of the protein was correlated with the number of citrullinated arginines. As citrullination of arginine abrogates the site of trypsin cleavage, the number of digested peptides diminishes with the rate of citrullination. This was expressed in mass spectra whose peak scarcity was related to the isoelectric point value of citrullinated aldolase (data not shown).

The modification of the isotopic mass cluster linked to citrullination is particularly well illustrated by the peptide corresponding to (RLQSIGTENTEENR) of 1,646.81 Da (theoretical mass). For spot 1, the isotopic cluster was classic with a first peak that appears at 1,646.85 Da (Figure 3c). For spot 5, the isotopic cluster is modified since the first peak is less intense than the second one, which appears at 1,647.70 kDa, in relation to its citrullination (Figure 3d). It is noteworthy that all these observations are valid for *in vitro *aldolase citrullination and can be extended to the HL-60 cell extract as well.

### Fourier transform ion cyclotron resonance mass spectrometer

After digestion of the spots by trypsin, in-gel nano-liquid chromatography–MS/MS analysis was performed on a nano-ESI-Q-FT-ICR instrument (Model: Apex Q-e, Bruker, Bremen, Germany) with the quadrupole analyzer set at the fixed mass *m/z *824, a mass window of *m/z *± 2 and a collision energy of 28.5 eV. A major peak was found in each gel spot. The mass of the parent ion was ascertained from liquid chromatography/MS performed at 1.5 eV collision energy and the quadrupole not resolving in Radio-Frequency-only mode.

The three peaks are discharged ions at *m/z *823.910, *m/z *824,401 and *m/z *824,403 respectively. The first peak therefore corresponds to a native peptide, whereas the second and the third peaks, a mass unit higher, are deamidated or citrullinated. Unfortunately these are two peptides with exactly the same mass corresponding to the sequences 43 to 56 RLQSIGTENTEENR and 44 to 57 LQSIGTENTEENRR, which differ only by the position of the R residue either at the N-terminal or C-terminal position (theoretical *m/z *823.908 for the native peptide, and theoretical *m/z *824.403 for the deaminated or citrullinated).

Inspection of the MS/MS spectra allows ascertaining the sequences since a long y series is present on each MS/MS spectrum (see Table 4). The first peak may therefore be attributed to a mixture of native LQSIGTENTEENRR and RLQSIGTENTEENR. The second peak to LQSIGTENTEE(NRR) bears a deamidation or citrullination on the NRR sequence. As the first y ion detected is y_3_, the precise position and therefore the nature of the modification cannot be ascertained. We were pleased that the third peak may be unambiguously assigned to RLQSIGTENTEENR bearing a citrullination on the R residue on the N-terminal side. Finally, we must point out that other citrullinated peptides have been identified corresponding to the sequence RALANSLACQGK (sequence 331 to 342).

**Table 4:** Fourier transform ion cyclotron resonance spectra of citrullinated aldolase

	Peptide sequence^a^				Peak 1a	Peak 1b	Peak 2	Peak 3
					
	Sequence 1	Sequence 2	Sequence 3	Sequence 4				
y1	175.119	175.119	175.119	175.119				
y2	331.220	331.220	289.162	289.162				
y3	445.263	446.247	418.204	418.204		445.269	446.247	418.197
y4	574.306	575.290	547.247	547.247	574.312	574.312	575.280	547.248
y5	703.348	704.332	648.295	648.295	648.294	703.347	704.336	648.297
y6	804.396	805.380	762.338	762.338	762.336		805.381	762.340
y7	918.439	919.423	891.380	891.380	891.386	918.455	919.425	891.391
y8	1,047.481	1,048.465	992.428	992.428	0.000		1,048.472	0.000
y9	1,148.529	1,149.513	1,049.449	1,049.449	1,049.449		1,149.496	1,049.454
y10	1,205.551	1,206.535	1,162.533	1,162.533	1,162.529		1,206.535	1,162.535
y11	1,318.365	1,319.619	1,249.565	1,249.565	1,249.563		0.000	1,249.569
y12	1,405.667	1,406.651	1,377.264	1,377.264			1,406.656	
y13	1,533.725	1,534.709	1,490.708	1,490.708				

^a^Sequence 1, LQSIGTENTEENRR; Sequence 2, LQSIGTENTEE(N)RR; Sequence 3, RLQSIGTENTEENR; Sequence 4, (R)LQSIGTENTEENR.

### Detection of citrullinated proteins on two-dimensional gel electrophoresis protein maps

**Figure 4:**
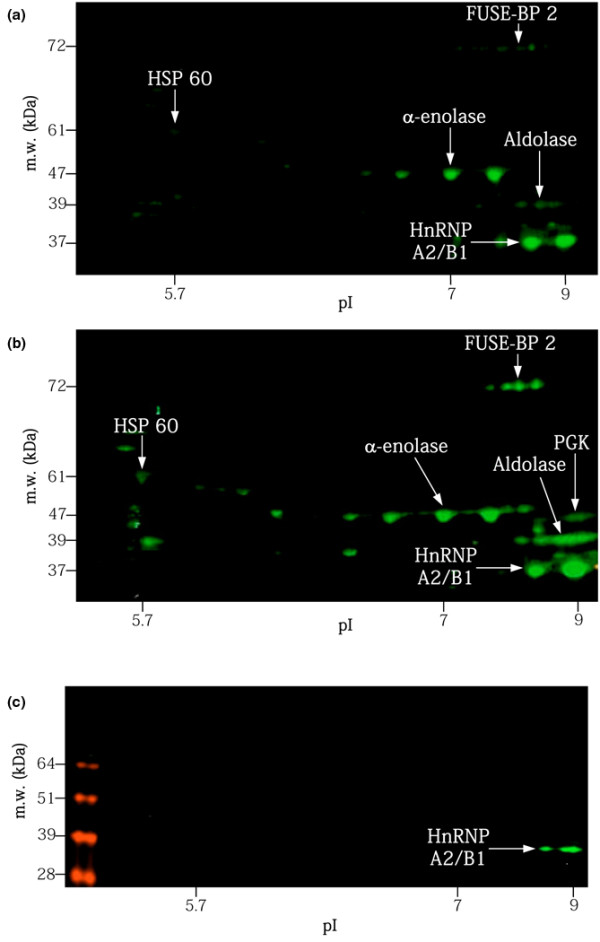
Detection of citrullinated proteins on two-dimensional gel electrophoresis HL-60 protein maps. Citrullinated proteins were detected on two-dimensional gel electrophoresis HL-60 protein maps **(a) **before and **(b) **after treatment with peptidyl-arginine deiminase. The membranes were incubated with rabbit anti-citrulline antibodies, washed and then incubated with biotinylated-goat anti-rabbit antibodies followed by IRDye 800-conjugated streptavidin, and were visualized using the Odyssey™ Infrared Imaging system. FUSE-BP, far-upstream element-binding protein; HnRNP A2/B1, heterogeneous nuclear ribonucleoprotein A2/B1; HSP60, 60 kDa heat shock protein; m.w., molecular weight; PGK, phosphoglycerate kinase 1.

Differentiated HL-60 cells have been previously shown to express PADI [24,25]. To assess the presence of citrullinated peptides in HL-60-derived proteins, we used anti-citrulline antibodies to immunoscreen HL-60 protein maps by western blot analysis. On the replicas of these maps, several spots were consistently detected by anti-citrulline antibodies (Figure 4a); in particular, spots previously characterized as α-enolase, aldolase and, at a lower level, HSP60 and FUSE-BP2. In another set of experiments, HL-60 protein maps were incubated with PADI for one night at 37°C. On these PADI-treated membranes, HSP60 and FUSE-BP2 were brighter and PGK1 was also revealed by anti-citrulline antibodies, suggesting that it effectively possesses citrullination sites (Figure 4b). It could be noted that the spots corresponding to heterogeneous nuclear ribonucleoprotein A2/B1 reacted with conjugate alone and represent a false positive reaction (Figure 4c).

### RA autoantibody reactivities against newly created citrullinated peptides

To confirm the antigenic structure that was targeted by autoAb present in RA sera, we analyzed their reactivity against the citrullinated peptides identified by MALDI-TOF MS analysis on the different deiminated proteins (aldolase, α-enolase, PGK1, HSP60, FUSE-BP1 and FUSE-BP2), although it was expected that not all identified sequences described in Table 3 were, or carried, B-cell epitopes. Interestingly, we noticed a significant association between the presence of anti-p46 antibodies and the reactivity against the peptide derived from α-enolase (*P *= 0.0047), between the presence of anti-p62 and reactivity against HSP60 peptide (*P *= 0.016), and between the presence of anti-p67 and reactivity against FUSE-BP2 peptide (*P *= 0.04), which confirms the identities of these polypeptides and may suggest that they could represent antigenic determinants recognized by RA autoAb.

**Figure 5:**
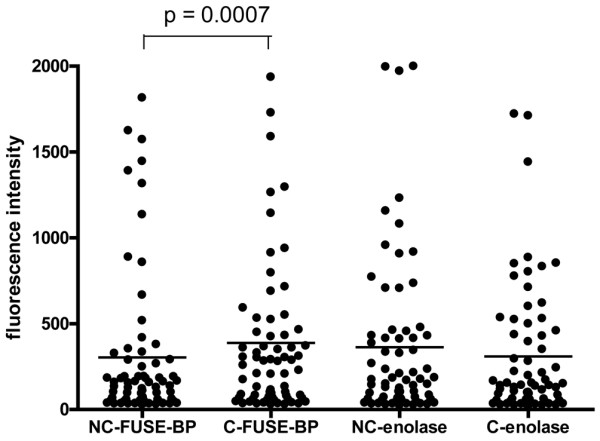
IgG response of rheumatoid arthritis sera to noncitrullinated and citrullinated linear peptides. The IgG response of the 110 rheumatoid arthritis sera to noncitrullinated (NC) and citrullinated (C) linear peptides was detected using Luminex technology. These peptides derived from α-enolase (YNQLL**R_citr_**IEEELGSKAK) and from far upstream element-binding protein (FUSE-BP) (VPDGMVGFIIG**R_citr_**GGEQISR). FUSE-BP, far-upstream element-binding protein.

With this regard, additional experiments were performed to confirm that the antigenicity of the selected peptides was due to the presence of citrulline. We focused on two peptides, the first (YNQLL**R_citr_**IEEELGSKAK) derived from α-enolase and the second from the FUSE-BP proteins, a peptide similar to FUSE-BP1 and FUSE-BP2 that is certainly the most interesting candidate autoantigen since the others were previously shown to be recognized by RA sera. In this respect, we compared the reactivity of RA sera with citrullinated and noncitrullinated α-enolase and FUSE-BP linear peptides. The results shown in Figure 5 clearly indicate that antigenicity of the FUSE-BP peptide is highly dependent on citrullination, while there was no difference concerning the reactivity against the native and citrullinated forms of the α-enolase peptide.

### Relationship between ACPA, reactivity pattern against proteins and peptide binding

Since the ACPA assay is thought to detect most antibodies directed against citrullinated peptides, we expected to find a significant association between the titers of ACPA and the presence of autoAb directed against 1-DE bands corresponding to citrullinated proteins. A significant association was therefore observed between ACPA titers and the presence of autoAb, which respectively bound to p39 (*P *= 0.02), to p46 (*P *= 0.05), to p58 (*P *= 0.04) and to p62 (*P *= 0.03).

We observed that ACPA presence or absence in RA serum at inclusion, however, was not totally correlated to the reactivity directed against the citrullinated peptides. Indeed, among the 36 RA sera that were positive for ACPA at inclusion, 20 of the sera did not possess any autoAb directed against the citrullinated peptides. Conversely, among the 74 RA sera that were negative for ACPA at inclusion, we observed that 18 (24%) of the sera possessed autoAb against FUSE-BP peptide. A total of 54 patients, from the 110 diagnosed as having RA after 2 years of follow-up, were therefore positive at inclusion either for CCP2 ELISA or for FUSE-BP-derived peptide, thus giving a percentage of 49% of ACPA-positive patients.

## Discussion

The objective of the present study was to identify new autoantibody markers in RA. For this purpose, we characterized the antigens targeted by autoAb present in sera obtained from early untreated RA patients, using 1-DE-separated and 2-DE-separated HL-60 cell extracts followed by in-gel proteolytic digestion and MALDI-TOF mass spectrometric analysis.

Ten proteins were shown to be frequently recognized by RA antibodies and were subsequently identified. Six of these proteins corresponded to already-described RA antigens – heterogeneous nuclear ribonucleoprotein A2/B1 [26,27], aldolase [28], α-enolase [29], calreticulin [30,31], BiP [32,33] and HSP60 [34,35] – demonstrating the validity of our methodology approach. Four other proteins – PGK1, stress-induced phosphoprotein 1 and FUSE-BP1 and FUSE-BP2 – constitute new candidate RA autoAb targets. A detailed analysis of MS spectra enabled us to show that seven of these antigens contain potentially deiminated peptides: aldolase, α-enolase, PGK1, calreticulin, HSP60, FUSE-BP1 and FUSE-BP2. Western blot analysis confirmed the presence of such residues in aldolase, α-enolase, HSP60 and FUSE-BP1 and confirmed the ability of another autoantigen, PGK1, to be citrullinated *in vitro*.

A significant association was observed between ACPA positivity and titer and the reactivity of RA sera against p39, p46, p58 and p62, which indirectly argues for the involvement of antibodies directed against citrulline-containing sequences in these anti-polypeptide reactivities. These data led us to analyze the reactivity against noncitrullinated peptides and citrullinated peptides derived from the different proteins, and our interest was focused on two peptides derived from α-enolase and FUSE-BP with the hypothesis that these two citrullinated peptides may represent new antigenic determinants. Firstly, the reactivity of RA sera against the α-enolase peptide selected by MALDI-TOF data is not related to citrullination. This result is not surprising since this peptide (*YNQLL****R***_***citr***_*IEEELGSKAK*) is not an immunodominant citrullinated epitope recognized by autoAb directed against citrullinated α-enolase. Indeed, in a recent report Lundberg and colleagues have demonstrated that the RA antibody response to human citrullinated α-enolase is directed against an immunodominant peptide (peptide 1A), different from that identified in the present study and one that cross-reacts with that recognizing *Porphyromonas gingivalis *enolase [36]. Interestingly, our different experiments suggest that a citrullinated peptide derived from the FUSE-BPs constitutes a new B-cell epitope of the RA autoimmune response. Indeed, the role of citrulline in the anti-FUSE-BP antibody response was demonstrated herein by the lower reactivity to the arginine-containing control peptide. Furthermore, the citrulline residue of this peptide is flanked by glycine amino acids that have been shown to enhance ACPA recognition and binding like serine residues [37]. Pertinently, this citrullinated FUSE-BP peptide provide additional information to ACPA since 24% of very early RA patients negative for ACPA possessed autoAb against this peptide. Further studies are needed to replicate these results but they do suggest that this autoAb population may enhance sensibility of the current anti-CCP2 test.

Citrullinated vimentin has been shown to be targeted by RA autoimmune response and to be a part of the Sa system [9,38]. Furthermore, the interest of antibodies to mutated citrullinated vimentin for diagnosing rheumatoid arthritis in anti-CCP-negative patients and for monitoring infliximab therapy was recently suggested [39]. In our study, we did not find any antibodies against citrullinated vimentin. Unmodified vimentin is therefore present in our HL-60 protein map and was identified with MS at a MW of 53 kDa and an isolectric point of 5.1 (data not shown). Using our HL-60 cell extract, however, vimentin was not detected by rabbit anti-citrulline antibodies. The lack of citrullination of the vimentin contained in the HL-60 cell extract might thus explain why citrullinated vimentin was not identified in our study as a target of early RA patient sera.

This proteomic approach allows one to demonstrate, on the one hand, that the autoAb response in early untreated RA is also directed against two categories of antigens, some enzymes of the glycolytic family and chaperones; and, on the other hand, that citrullination is involved in the antigenic properties of some of these antigens. The glycolytic enzyme family appears to play an important role in the autoimmune response observed in both RA and the RA experimental model. Indeed, aldolase and α-enolase have been already described as autoantigens in RA [28,29]; and in the K/BxN mouse model, arthritis is induced by pathogenic autoAb directed against another glycolytic enzyme, the glucose-6-phosphate isomerase [40]. The role of citrullination in the recognition of α-enolase by RA patient-derived autoAb has been reported [10]. In addition, our study indicates that both aldolase and PGK1 also contain deiminated peptides, suggesting that the recognition of glycolytic enzymes by RA sera is associated with their potential to undergo this post-translational modification.

Calreticulin, HSP60 and BiP are molecular chaperones promoting folding and oligomeric assembly of newly synthesized polypeptides in endoplasmic reticulum [41]. They also constitute targets of the autoimmune response in RA and systemic lupus erythematosus [42]. Stress-induced phosphoprotein 1 (also called Hop) acts as an adaptor that directs heat shock protein 90 to heat shock protein 70 protein complexes and can modulate the chaperone activities of these heat shock proteins [43].

c-*myc *is a proto-oncogene considered a master regulator of cell proliferation, growth, differentiation, senescence and death [44]. The far-upstream element of the human c-*myc *gene stimulates promoter activity when bound by a trans-acting protein [45] such as members of the FUSE-BP family [46]. These nuclear proteins are expressed in proliferating cells of various lineages but not in quiescent cells [45,47]. In RA, synovial tissue fibroblast hyperplasia is reminiscent of tumor-like proliferation, and fibroblasts exhibit elevated gene expression of proto-oncogenes such as c-*myc *[48,49] – suggesting that the FUSE-BPs could be overexpressed in these cells, which could lead to the production of autoAb. Citrullination of proteins is mediated by PADI, which converts peptidylarginine into citrulline. It is thought to occur under extreme conditions, such as stress, apoptosis [50,51] and inflammation [52], and then to inactivate the function of a protein as shown by *in vitro *experiments. In this regard, Sawada and colleagues showed that the tumor suppressor p53, present in a citrullinated form in RA synovial fibroblasts, loses its function and may participate in the tumor-like growth of RA synovium [53]. Citrullination of the FUSE-BPs, by deregulating c-*myc*, might then contribute to the RA synovial hyperplasia.

## Conclusions

The presented proteomic approach shows that two categories of antigens, enzymes of the glycolytic family and molecular chaperones are also targeted by the early untreated RA autoAb response. For some of these, and notably the FUSE-binding proteins, citrullination is involved in the immunological tolerance breakdown observed earlier in RA patients. Our results also point out that autoAb recognizing a citrullinated peptide from FUSE-BP may enhance the sensibility for RA of the current available anti-CCP2 test. An extensive study of the prevalence of antibodies directed against peptides derived from these new autoantigens is underway to precisely determine their predictive values in RA diagnosis and prognosis.

## Abbreviations

1-DE: one-dimensional gel electrophoresis; 2-DE: two-dimensional gel electrophoresis; ACPA: anti-citrullinated protein antibodies; autoAb: autoantibodies; CCP: cyclic citrullinated peptide; DTT: dithiothreitol; FCS: fetal calf serum; FT-ICR: Fourier transform ion cyclotron resonance; FUSE-BP: far-upstream element-binding protein; HSP60: 60 kDa heat shock protein; MALDI-TOF: matrix-assisted laser desorption/ionization–time of flight; MS: mass spectrometry; MW: molecular weight; PADI: peptidylarginine deiminase; PBS: phosphate-buffered saline; PGK: phosphoglycerate kinase; PTM: post-translational modification; RA: rheumatoid arthritis; VErA: Very Early Arthritis.

## Competing interests

The authors declare that they have no competing interests.

## Authors' contributions

VG, MT-L'O, FT, OV and DG conceived and carried out the study and drafted the manuscript. RC carried out the MS work. RD performed the statistical analysis. PF and XLL participated in the recruitment of the patients and helped in the design of the study. All authors read and approved the final manuscript.
